# Ovarian cycle activity varies with respect to age and social status in free-ranging elephants in Addo Elephant National Park, South Africa

**DOI:** 10.1093/conphys/cot025

**Published:** 2013-10-18

**Authors:** Elizabeth W. Freeman, Jordana M. Meyer, Sarah B. Putman, Bruce A. Schulte, Janine L. Brown

**Affiliations:** 1 New Century College, George Mason University, Fairfax, VA 22030, USA; 2 Center for Species Survival, Smithsonian Conservation Biology Institute, Front Royal, VA 22630, USA; 3 Department of Biology, Western Kentucky University, Bowling Green, KY 42101, USA

**Keywords:** *Loxodonta africana*, matriarch, non-invasive hormone monitoring, population management, post-partum duration, post-reproductive lifespan

## Abstract

Free-ranging African elephants live in a fission–fusion society, at the centre of which is the matriarch. Matriarchs are generally older females that guide their families to resources and co-ordinate group defense. While much is known about elephant society, knowledge is generally lacking about how age affects the physiology of wild elephants. Investigation of the ovarian activity of free-ranging elephants could provide insight into the reproductive ageing process, with implications for population management. Faecal samples were collected from 46 individuals ranging in age from 14 to 60 years for a 2-year period, and progestagen metabolite analyses were used to examine relationships between social status, age, season, and ovarian activity in female elephants in Addo Elephant National Park, South Africa. Social status was the strongest predictor of faecal progestagen metabolite concentrations in non-pregnant elephants, with grand matriarchs (*n* = 6) having the lowest values compared with matriarchs (*n* = 21) and non-matriarch females (*n* = 19). Likewise, social status and age were the strongest predictors of faecal progestagen metabolite concentrations in pregnant elephants (*n* = 27). The number of years since a non-pregnant female gave birth to her last calf (post-partum duration) was longer for older females with a higher social status, as well as during the dry season. Our results indicate that social standing and age of elephants are related to reproductive function, and that older females exhibit reductions in ovarian capacity. These results expand our understanding of reproduction and fertility throughout an elephant's lifespan, and the factors that impact gonadal function in free-ranging females. Given that possible over-abundance of elephants in areas such as Addo Elephant National Park is fuelling the debate over how best to manage these populations, knowledge about the reproductive potential of high-ranking females can provide managers with biological data to identify the best candidates for controlling growth through translocation or contraception.

## Abstract (precis)

We investigated reproductive aging in African elephants and found a relationship between females obtaining a high social status within their herd and a decline in ovarian steroid activity, which may be analogous to menopause. Understanding possible social constraints on reproductive fitness could enhance management of aging and growing elephant populations.

## Introduction

In the wild, African elephants (*Loxodonta africana*) live in a fission–fusion society (Archie *et al.*, 2006), and adult females and their offspring form the basis of the family unit (Douglas-Hamilton, 1972; Dublin, 1983; Archie *et al.*, 2006). Females remain with the family group throughout their lives, whereas males leave their natal group at between 12 and 15 years of age (Poole, 1994). The largest, eldest female in the family is usually the matriarch (Douglas-Hamilton, 1972; Poole *et al.*, 1989; Archie *et al.*, 2006). However, age alone does not guarantee matriarchal status, because at the death of a matriarch the family can fission and may not always follow the next oldest female (B. A. Schulte, personal observation). When multiple, related family groups fuse into a kinship group, the eldest matriarch (which we term the ‘grand matriarch’) generally adopts the leadership role (Wittemyer *et al.*, 2007c).

The importance of the matriarch is as a leader with crucial knowledge of natural resources and as a co-ordinator of group defense (Douglas-Hamilton, 1972; Dublin, 1983; Poole and Moss, 1989; Esposito, 2008; McComb *et al.*, 2011). How she achieves this coordination is uncertain, but a matriarch seems to be aware of the location of family/kinship and non-family/kinship members, or the difference between relatives and non-relatives (Soltis *et al.*, 2005a, b; Bates *et al.*, 2008). The interactions of matriarchs and their families with related and unrelated groups may result in amiable fusion, tolerance, avoidance, or aggression (Esposito, 2008). Older matriarchs are more successful at distinguishing intruders (McComb *et al.*, 2001) and the calls of male lions (McComb *et al.*, 2011), and they facilitate family success in extreme conditions, such as severe poaching (Gobush *et al.*, 2008). Age also affects a matriarch's rank among other matriarchs and the relative rank of her family; matriarchal rank contributes to the dominance status of non-matriarchal females in her kinship group in comparison with females from other kinship groups (Wittemyer and Getz, 2007c).

A positive relationship between social status and age has also been documented in a number of other species, yet knowledge is generally lacking about the biology of ageing and the pathological processes that sometimes accompany advanced age (Erwin *et al.*, 2008). This lack of knowledge may hinder successful management, propagation, and conservation of endangered species, which depends upon a detailed understanding of reproduction and fertility throughout the lifespan (Erwin and Hof, 2008). In most species, gradual, age-related decreases in physiological functions occur in the majority of individuals and typically coincide with somatic and reproductive senescence (Kachel *et al.*, 2011). Declines in fertility with age are a common feature of mammal life histories, particularly for long-lived species with long reproductive lifespans and inter-birth intervals (Bellino *et al.*, 2003; Emery Thompson *et al.*, 2007), such as chimpanzees (Emery Thompson *et al.*, 2007), killer whales (McAuliffe *et al.*, 2005; Johnstone *et al.*, 2010), and free-ranging elephants (Laws *et al.*, 1970; Smuts, 1975; Moss, 2001; Freeman *et al.*, 2009; Robinson *et al.*, 2012). Furthermore, African elephants in zoos exhibit a decline in ovarian activity that is associated with a high social rank and in some, but not all instances, a more advanced age (Freeman *et al.*, 2010b). Hermes *et al.* (2004) referred to the decline in zoo elephant reproductive function as asymmetric or premature reproductive ageing. The rate of ageing and reproductive senescence is influenced by trade-offs in life-history and environmental variation, which can explain the differences in longevity observed within and between species (Nussey *et al.*, 2008). It is not clear whether elephants exhibit these same trade-offs, or if age and social rank have similar suppressive effects on reproductive function.

Environmental factors also can impact reproductive success in free-ranging African elephants. Females can breed year round; however, conception and birth rates show strong seasonality relative to precipitation (Laws, 1970; Smuts, 1975; Dublin, 1983; Gough *et al.*, 2006; Wittemyer *et al.*, 2007a; Freeman *et al.*, 2009; Foley *et al.*, 2010). A few studies have investigated the impact of ecological factors on faecal progestagen metabolite (FPM) concentrations in free-ranging elephants as an indicator of reproductive activity (Whitehouse *et al.*, 2000; Foley *et al.*, 2001; Wittemyer *et al.*, 2007b; Gobush *et al.*, 2008). For example, poor vegetative quality during the dry season in Northern Kenya is correlated with lower FPM levels (Wittemyer *et al.*, 2007b). Likewise, seasonal declines in FPM concentrations occur during the dry seasons in Tanzania, when water availability and food quality affect the body condition of the females (Foley *et al.*, 2001). It is not known whether other populations of African elephants have similar seasonal variability in FPM concentrations, or how the ageing process impacts ovarian activity. Expanding our understanding of the reproductive physiology of free-ranging elephants in the presence of multiple environmental stressors would enhance our ability to evaluate and improve the efficacy of various conservation and management practices (Cooke *et al.*, 2013).

The goal of the present study was to investigate relationships among female age, social status, and seasonal variability in precipitation with FPM concentrations in free-ranging elephants of Addo Elephant National Park (AENP). We hypothesized that low FPM concentrations would correlate inversely with female age, social status, post-partum duration, and seasonal precipitation. Understanding how elephant reproductive success is related to age and social status would contribute to the growing field of conservation physiology (Cooke *et al.*, 2013) and broaden our understanding of the ageing process, potentially aiding the reproductive management of free-ranging and zoo elephants, and perhaps other long-lived species.

## Materials and methods

### Study animals

**Figure 1:**
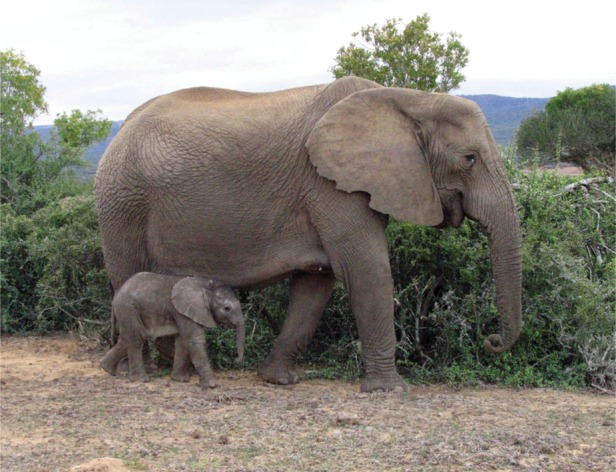
Non-matriarch and her calf. First sighting of a female African elephant in Addo Elephant National Park, South Africa with her newborn calf. She was identified as HAN by the distinctive notches in her ears and association with the rest of her family group (not shown).

The study site was in the Eastern Cape of South Africa at Addo Elephant National Park, which consists of 13 500 ha of habitat that ranges from sub-tropical succulent thicket to open, grassy plains (Whitehouse and Hall-Martin, 2000). There were ∼415 elephants, comprising six matrilines, in AENP. Elephants were identified using ear tears and vein patterns, as well as other physical features (Fig. 1) that were compared with files consisting of photographs and descriptions of the individuals (Whitehouse and Hall-Martin, 2000; Whitehouse *et al.*, 2001a). The date of birth was estimated to the month and year based upon photographic data from 1976 to the present (Whitehouse and Hall-Martin, 2000). For the younger females, the timing of the birth was known within a day or week of the event, based on observations (Whitehouse and Hall-Martin, 2000; Loizi *et al.*, 2009). For females born before 1976, age was estimated using well-documented patterns of change in physical characteristics (e.g. shoulder height and body shape) relative to age in free-ranging elephants (Moss, 2001; Wittemyer *et al.*, 2007a). Longitudinal studies of marked or recognizable individuals, such as those in AENP, are the most reliable sources for information about reproductive senescence in wild populations, because they allow researchers to separate within-individual ageing patterns from between-individual heterogeneity (Nussey *et al.*, 2008).

### Behavioural observations and faecal ­samples

The project was conducted from July 2007 to September 2009. In-depth analyses of AENP data since 1931 have established that there are six elephant matrilines in the park that form six kinship groups and two clans (Whitehouse and Harley, 2001). Additionally, 25 family groups, each with an identifiable matriarch, have been discovered through detailed field studies of the AENP population (from 1996 to 2009; Bagley, 2004; Gough and Kerley, 2006; Meyer, 2006; Esposito, 2008; Loizi *et al.*, 2009; Merte *et al.*, 2010). Given that the social rank of adult African elephants varies within and between matrilines (Wittemyer and Getz, 2007c), efforts were made to observe post-pubertal females (46 elephants; range, 14–60 years of age; Table 1) within each social category from all six of the AENP matrilines. The social status of each female within her matriline was assigned based upon the following criteria. Grand matriarchs (*n* = 6; age range, 39–60 years at the start of the study) were the head of their kinship group during fusion events. Matriarchs (*n* = 21; age range, 22–46 years) were the remaining heads of their respective family units during fission events. Non-matriarchs (*n* = 19; age range, 13–33 years) were the remaining females within the kinship group that did not assume a leadership role during fusion or fission events.

**Table 1:** Description of the female African elephants studied in Addo Elephant National Park and faecal samples collected from each individual

Elephant	Social status	Kinship group	Family group^a^	Age range (years)^b^	Total faecal samples	Pregnant faecal samples
AND	Grand matriarch	A	AND	51–53	16	0
ALO^c^	Matriarch	A	ALO	46–48	15	0
LAG^c^	Matriarch	A	LAG	44–46	14	0
AMA^c^	Matriarch	A	AMA	38–40	17	7
ALL^c^	Matriarch	A	ALL	35–37	19	5
APP^c^	Matriarch	A	APP	33–35	19	10
AMB^c^	Matriarch	A	AMB	30–32	13	0
ANN^c^	Matriarch	A	ANN	22–24	16	8
ANG^d^	Non-matriarch	A	AND	28–30	11	0
ARR	Non-matriarch	A	LAG	26–28	18	13
ARA	Non-matriarch	A	ALL	19–21	16	0
AMO	Non-matriarch	A	AMA	17–18	7	5
TAN	Grand matriarch	B	TAN	56–58	18	0
CAT	Matriarch	B	CAT	37–39	23	12
BEV^d^	Matriarch	B	BEV	37–39	16	12
BLU^c^	Matriarch	B	BLU	31–33	19	14
BON	Matriarch	B	BON	27–29	24	21
BCH	Non-matriarch	B	BCH	33–35	17	12
BUB	Non-matriarch	B	BEV	24–26	17	6
BWI	Non-matriarch	B	CAT	17–19	17	8
BUL	Non-matriarch	B	CAT	14–15	6	4
HET	Grand matriarch	H	HET	57–59	16	0
HEI^d^	Matriarch	H	HET	35–37	14	0
HIL^d^	Non-matriarch	H	HET	31–33	13	11
HAN^d^	Non-matriarch	H	HET	26–28	14	10
LLT	Grand matriarch	L	LLT	39–41	7	5
LAU	Matriarch	L	LAU	35–37	5	0
LUC	Non-matriarch	L	LAU	22–24	5	4
AFS	Grand matriarch	P	AFL	58–60	12	0
MAR^c^	Matriarch	P	MAR	44–46	13	0
MEG^c^	Matriarch	P	MEG	42–44	9	0
PAU^d^	Matriarch	P	PAU	38–40	13	0
MAN	Matriarch	P	MAN	35–36	7	0
TIP	Matriarch	P	TIP	34–36	14	6
PHY	Matriarch	P	PHY	26–28	21	0
MOL	Matriarch	P	MAR	26–28	18	0
MUS	Non-matriarch	P	MEG	24–26	12	10
PIP	Non-matriarch	P	PAU	22–24	12	4
MIR	Non-matriarch	P	MEG	18–20	17	0
POP	Non-matriarch	P	PAU	18–20	13	2
MIL	Non-matriarch	P	MAR	13–15	10	0
REB	Grand matriarch	R	REB	43–45	11	4
ROZ	Matriarch	R	ROZ	32–34	16	4
RHO^d^	Non-matriarch	R	REB	30–32	12	7
RHI	Non-matriarch	R	ADD	28–29	8	4
RHE	Non-matriarch	R	RHO	14–15	6	1

^a^Family group was designated by the matriarch, or grand matriarch, of the group.

^b^Age range designates the age of the elephant over the course of faecal sample collections.

^c^Sister of the grand matriarch.

^d^Daughter of the grand matriarch.

Addo Elephant National Park elephants have been monitored intensively since 1996 (Whitehouse and Hall-Martin, 2000) and are habituated to the presence of research and tourist vehicles, which made it easy to observe their behaviour and collect faecal samples from known individuals. During focal observations of the 46 females, reproductive events, including oestrous behaviours, mate guarding by males and copulations (Moss, 1983; Wittemyer *et al.*, 2007b), and the development of teats were recorded. Pregnancy (*n* = 27 females; age range, 14–45 years) was determined according to these events and by back-dating parturition events. The date of conception was calculated by subtracting the average gestation period of 22 months (Laws, 1969; Wittemyer *et al.*, 2007b) from the estimated date of birth. Based upon parturition events, we were able to confirm that two grand matriarchs (33.3%), 10 matriarchs (50.0%) and 15 non-matriarchs (78.9%) were pregnant during the course of this study. Faecal samples were collected when elephants were observed defaecating to ensure proper identification, and as close to monthly from each individual as possible. We analysed 636 faecal samples (13.83 ± 0.18 per individual; Table 1); 209 of these were from pregnant individuals (*n* = 27 elephants), while the remaining 427 were from non-pregnant animals (*n* = 46 elephants).

Our methods adhered to the Association for the Study of Animal Behaviour/Animal Behavior Society *Guidelines for the Use of Animals in Research* and have been approved by the Institutional Animal Care and Use Committee (IACUC) of George Mason University (#A3210-01). The fieldwork was performed with the permission (Permit # 2002-12-11 BSCH) and support of personnel in the South African National Parks.

### Hormone analyses

A field method previously validated for elephants was used for extracting FPM (Freeman *et al.*, 2010a, 2011). One millilitre aliquots of faecal extracts were placed into 12 mm × 75 mm polypropylene tubes (#2332 and #2305; Perfector Scientific), air-dried and heated to 72°C for 30 min before shipment to the Smithsonian Conservation Biology Institute for analysis. Dried faecal extracts were reconstituted with buffer (0.2 M NaH_2_PO_4_ and 0.2 M Na_2_HPO_4_ in 0.14 M NaCl) by vortexing the tubes for 1 h and then sonicating for 30 min. Reconstituted extracts were diluted and analysed using the enzyme immunoassay methods of Graham *et al.* (2001), with a monoclonal progesterone antibody (1:10 000 dilution CL425; C. Munro, University of California-Davis, CA, USA), horseradish ­peroxidase-conjugated label (1:40 000 dilution; C. Munro) and a phosphate–citrate buffer (#P4560; Sigma Aldrich, Inc.) ­substrate with tetramethylbenzidine (#T3405; Sigma Aldrich, Inc.). Assay sensitivity was 0.78 pg/well, and intra- and inter-assay coefficients of variation were <10%.

### Data analyses

Given that FPM concentrations were higher in faeces from pregnant than non-pregnant elephants (Freeman *et al.*, 2011), samples were grouped for data analysis according to the reproductive state of the female at the time of sample collection. For non-pregnant elephants, the post-partum duration (PPD; i.e. the duration of the non-pregnant period) was determined by calculating the duration (in years) between the date of the sample collection and the parturition date of each elephant's previous calf (Wittemyer *et al.*, 2007b). The inter-pregnancy interval (IPI) was calculated for pregnant elephants as the duration (in years) between the birth of the elephant's previous calf and the conception of the current fetus (Wittemyer *et al.*, 2007b). As described previously, the month of gestation was determined by back-dating the average gestation period of 22 months (Laws, 1969; Wittemyer *et al.*, 2007b) from the estimated date of birth. Given that newborns may not have been detected on the exact date of birth, our estimates for month of gestation may vary by ± 1–2 weeks. Thus, we used the trimester of gestation (first, 0–7 months; second, 8–14 months; and third, 15–22 months; Duer *et al.*, 2002, 2007) to reflect the stages of pregnancy for model analyses. Total monthly precipitation values (in millimetres) for the AENP weather station were obtained from the South African Weather Service (Walmer, South Africa). Addo Elephant National Park is classified as semi-arid to arid and receives <455 mm precipitation per year on average (www.sanparks.org). Rainfall within AENP does occur throughout the year, but there are peaks in February–March and October–November. The AENP wet season was thus defined as October–March and the dry season as April–September.

Many of the explanatory variables were potentially correlated (e.g. the oldest elephants are most likely to be grand matriarchs). Thus, a linear mixed-effect (LME) model was employed to determine what factors contributed the most to FPM concentrations and PPD (Wittemyer *et al.*, 2007b). Mixed-effects models can control for sources of between-individual heterogeneity, thus allowing for more accurate measurement of within-individual ageing patterns in longitudinally measured life-history traits (Nussey *et al.*, 2008). Given that model data included repeated measures from individual elephants, female identity was incorporated as a random effect (Wittemyer *et al.*, 2007b). The LME model assumes a Gaussian (or normal) distribution of the variables. Normality of the data was tested using the Kolmogorov–Smirnov test, and those variables that were not normally distributed were transformed (e.g. square root or Log_10_) prior to inclusion in the model. Pregnancy FPM concentrations were examined with respect to the fixed effects of age, social status, fetal sex, month of gestation, IPI, monthly ­precipitation, and wet/dry season. In comparison, FPM concentrations from non-pregnant elephants were evaluated based on age, social status, PPD, sex of her previous calf, monthly precipitation, and wet/dry season. Lastly, PPD in non-­pregnant elephants was examined based on age, social status, sex of the previous calf, FPM concentrations, monthly precipitation, and wet/dry season; female identity was included as a random effect in the LME analyses of PPD. One elephant, MIR, was not included in the LME analyses because she had never given birth, and thus we could not calculate a PPD or provide a calf sex. Step-wise elimination of non-significant variables was conducted, and reduced models were compared with the full model using smaller values of Akaike's information criteria and Bayesian information criteria as a guide for model selection (Wittemyer *et al.*, 2007a; Freeman *et al.*, 2011).

Linear mixed-effects models were analysed using the free statistical package R (R Development Core Team, 2012) using the nlme package. When appropriate, *post hoc* analyses of the variables in the most parsimonious models were conducted using the HH package for Tukey's pair-wise comparisons. All other analyses were conducted using SigmaPlot (version 11.0 2008; Systat Software, Inc). For all analyses, *P* < 0.05 was considered significant, and all data were reported as means ± SEM, except for the LME tables, where the coefficient and standard error of the model were reported.

## Results

The average FPM concentration for samples collected from female African elephants in AENP was 107.45 ± 5.15 ng/g faeces, with FPM concentrations from pregnant animals being higher (130.88 ± 5.69 ng/g faeces) than those from non-pregnant females (95.93 ± 2.56 ng/g faeces; Table 2). The average IPI for pregnant elephants in AENP was 1.88 ± 0.55 years (range, 0.08–4.67 years). There was little variability in IPI with respect to social status of the elephant within her family (Table 2). In contrast, non-pregnant grand matriarchs had the greatest PPD, followed by matriarchs, and then non-matriarchs (Table 2). The average PPD for all non-pregnant elephants was 2.65 ± 0.71 years (range, 0.08–27.43 years).

**Table 2:** Physiological data collected from non-pregnant and pregnant free-ranging African elephants within Addo Elephant National Park, South Africa as they varied with respect to social rank of the individual within her family

	Non-pregnant elephants		Pregnant elephants	
Social rank	FPM (ng/g faeces)	PPD (years)	FPM (ng/g faeces)	IPI (years)
Grand matriarch	86.47 ± 5.01 (30.88–156.38)	6.30 ± 2.46 (0.67–17.06)	114.69 ± 27.08 (44.48–401.51)	1.29 ± 0.54 (0.75–1.83)
Matriarch	97.59 ± 3.21 (24.79–488.20)	2.99 ± 1.27 (0.31–27.43)	123.22 ± 6.82 (34.57–415.65)	2.08 ± 0.59 (0.08–4.33)
Non-matriarch	103.56 ± 5.81 (32.63–394.85)	1.03 ± 0.26 (0.04–4.00)	135.70 ± 4.36 (39.54–605.77)	1.84 ± 0.32 (0.08–4.67)

Data are presented as the mean value ± SEM and range (minimum–maximum) of faecal progestagen metabolite (FPM) concentrations, the number of years since non-pregnant females had their last calf (PPD), and the number of years between the conception of a pregnant female's current fetus and the birth of her previous calf (IPI).

**Figure 2:**
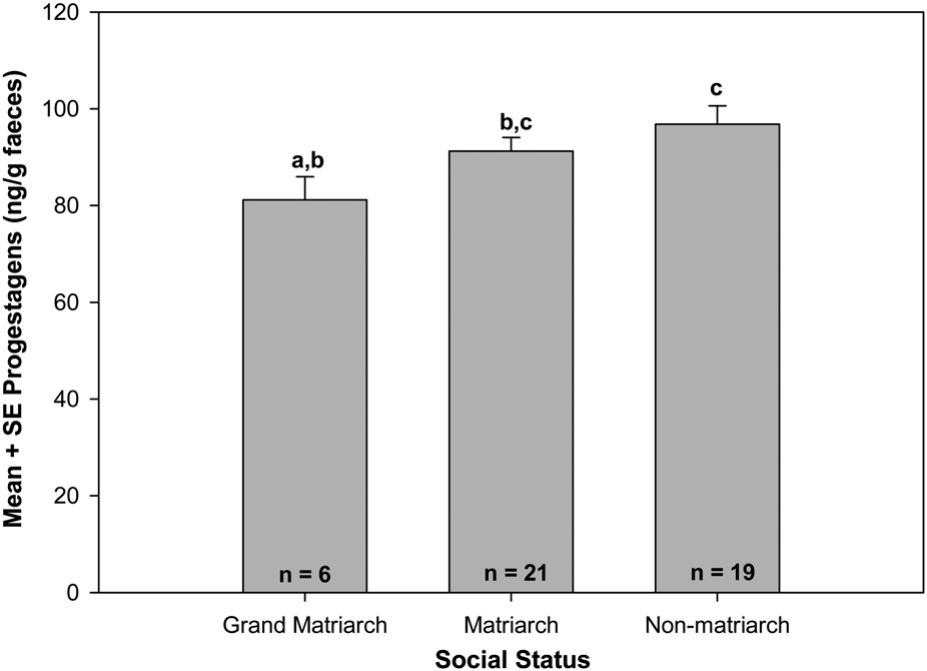
Faecal progestagens and pregnancy. Relationship between mean faecal progestagen metabolite concentrations and social rank for non-pregnant elephants in Addo Elephant National Park, South Africa. Superscripts designate significant differences (*P* < 0.05, Tukey's test).

Several variables impacted FPM concentrations in elephants within AENP. Due to the relationship between age and social status, we ran the full model with an interaction of the two. However, based upon values of Akaike's information criteria and Bayesian information criteria, the most parsimonious model to predict the FPM concentrations in non-pregnant elephants was a reduced model with no interaction term (Table 3). The only significant variable was social status of the female (*F*_2,41_ = 3.30, *P* = 0.05). The FPM concentrations for non-pregnant non-matriarchs were significantly higher than for non-pregnant grand matriarchs (Tukey's test, *P* = 0.03); however, no differences in FPM concentrations were found between non-pregnant matriarchs and non-pregnant grand matriarchs (Tukey's test, *P* = 0.06) or non-pregnant non-matriarchs (Tukey's test, *P* = 0.57; Fig. 2). The age of the female, the interaction of age and social status, PPD, total monthly precipitation at the time of sample collection, the sex of her last calf, and whether the sample was collected in the wet or the dry season did not significantly contribute to FPM concentrations in non-pregnant AENP elephants (Table 3).

**Table 3:** Linear mixed-effects model of factors that influence faecal progestagen metabolite concentrations in non-pregnant female African elephants in Addo Elephant National Park

Full model	Coefficient ± SEM	d.f.	*t*-value	*P*-value	Akaike's information criteria Bayesian information criteria
Intercept	61.34 ± 55.91	343	1.10	0.27	4035.82 4083.51
Age	0.53 ± 1.03	343	0.51	0.61	
Square root of PPD (years)	−0.75 ± 2.29	343	−0.33	0.74	
Log_10_ precipitation (mm)	−6.02 ± 5.87	343	−1.03	0.31	
Season (dry)					
Wet season	−5.55 ± 5.01	343	−1.11	0.27	
Sex of last calf (female)					
Last calf male	3.27 ± 4.24	40	0.77	0.45	
Social status (grand matriarch)					
Matriarch status	−28.68 ± 57.15	40	0.50	0.62	
Non-matriarch status	−38.96 ± 57.89	40	0.67	0.51	
Age × social status (grand matriarch)					
Age × matriarch status	−0.25 ± 1.08	343	−0.23	0.82	
Age × non-matriarch status	−0.37 ± 1.23	343	−0.30	0.77	
Reduced model	Coefficient ± SEM	d.f.	*t*-value	*P*-value	Akaike's information criteria Bayesian information criteria
Intercept	83.25 ± 4.98	348	16.73	<0.001	
Season (dry)					
Wet season	−7.45 ± 4.58	348	−1.63	0.10	
Social status (grand matriarch)					4007.85
Matriarch status	10.29 ± 5.55	41	1.85	0.07	4031.63
Non-matriarch status	15.04 ± 6.11	41	2.46	0.02	

**Figure 3:**
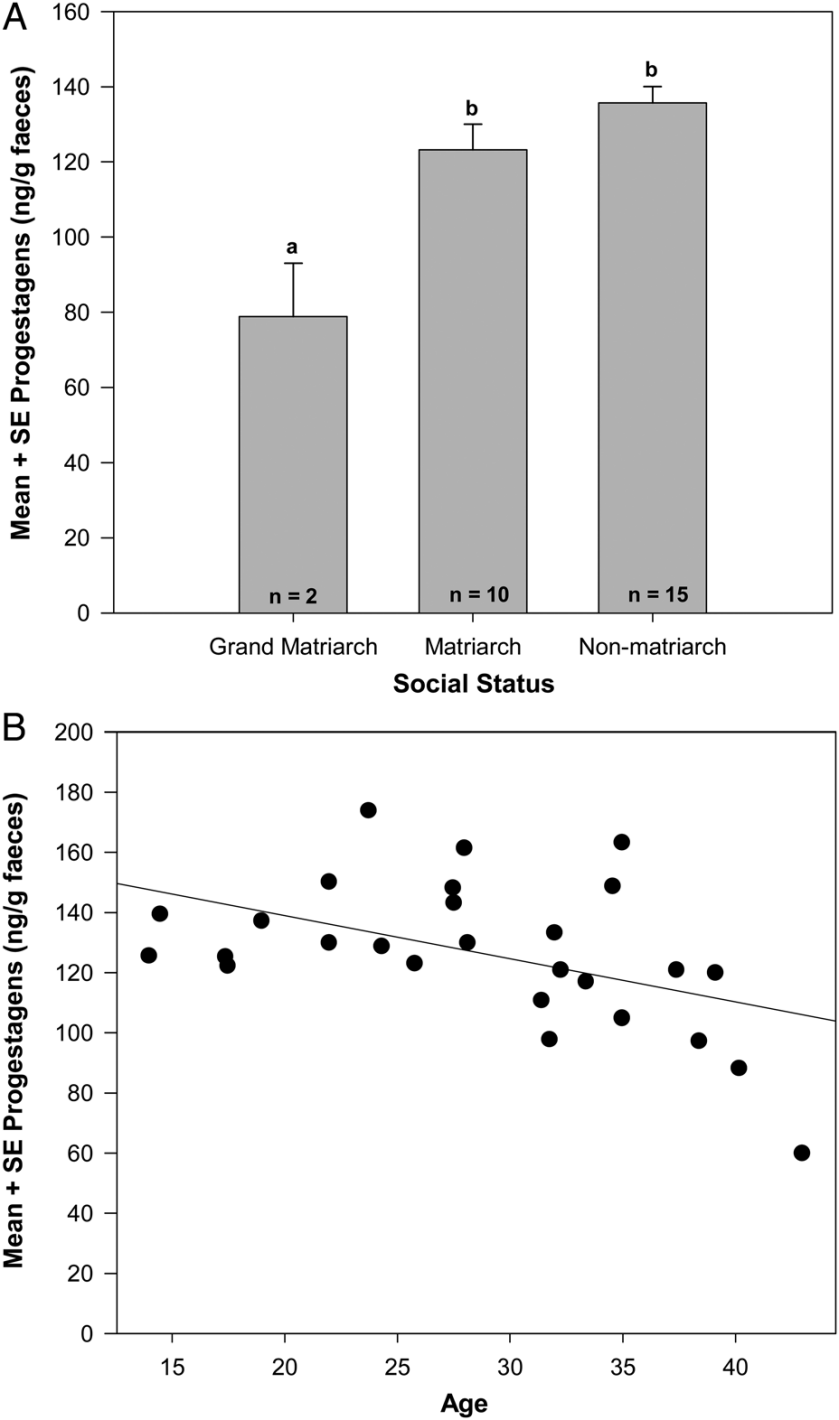
Faecal progestagens, social rank and age. Relationships between mean faecal progestagen metabolite concentrations and social rank (**A**) or age (**B**) of pregnant elephants in Addo Elephant National Park, South Africa. Superscripts designate significant differences (*P* < 0.05, Tukey's test).

In order to determine what factors contributed to FPM concentrations in pregnant elephants, we included the interaction of age and social status again. With these models, the interaction of age and social status impacted the FPM concentrations in pregnant elephants (Table 4), in both the full and the reduced models. Additionally, the sex of the fetus was significant in the reduced model, but had only a borderline relationship in the full model (Table 4). Based upon Akaike's information criteria and Bayesian information criteria, the full model was more parsimonious than the reduced model. Older, pregnant grand matriarchs had lower FPM concentrations than pregnant matriarchs (Tukey's test, *P* < 0.05) and pregnant non-matriarchs (Tukey's test, *P* < 0.05; Fig. 3A); no differences were found between older, pregnant matriarchs and pregnant non-matriarchs (Tukey's test, *P* = 0.41). While neither age (*F*_1,173_ = 0.29, *P* = 0.57) nor social status (*F*_1,24_ = 0.72, *P* = 0.50) alone contributed to FPM concentrations in pregnant AENP elephants, the interaction of age and social status was significant (*F*_2,173_ = 5.21, *P* < 0.01; Fig. 3B). None of the other variables (e.g. trimester, IPI, precipitation, season) contributed (*P* > 0.05) to FPM concentrations in pregnant AENP elephants (Table 4).

**Table 4:** Linear mixed-effects model of factors that influence faecal progestagen metabolite concentrations in pregnant female African elephants in Addo Elephant National Park

Full model	Coefficient ± SEM	d.f.	*t*-value	*P*-value	Akaike's information criteria Bayesian information criteria
Intercept	1852.02 ± 799.43	173	2.32	0.02	2359.64 2405.61
Age	−41.90 ± 19.37	173	−2.16	0.03	
Trimester (first)					
Second	25.31 ± 15.07	173	1.68	0.10	
Third	4.05 ± 15.00	173	0. 27	0.78	
IPI (years)	4.59 ± 5.47	173	0.84	0.40	
Log_10_ precipitation (mm)	−4.82 ± 15.90	173	−0.30	0.76	
Sex of fetus (female)					
Male fetus	−27.58 ± 15.15	173	−1.82	0.07	
Social status (grand matriarch)					
Matriarch status	−1625.96 ± 804.96	24	−2.02	0.05	
Non-matriarch status	−1816.64 ± 800.05	24	−2.27	0.03	
Season (dry)					
Wet season	7.46 ± 13.68	173	0.55	0.59	
Age × social status (grand matriarch)					
Age × matriarch status	39.01 ± 19.46	173	2.00	0.05	
Age × non-matriarch status	45.52 ± 19.24	173	2.37	0.02	
Reduced model	Coefficient ± SEM	d.f.	*t*-value	*P*-value	Akaike's information criteria Bayesian information criteria
Intercept	1605.07 ± 753.00	178	2.13	0.03	2387.55
Age	−35.60 ± 18.17	178	−1.96	0.05	2417.33
Sex of the fetus (female)					
Male	−1834.35 ± 791.50	178	−2.33	0.02	
Social status (grand matriarch)					
Matriarch status	−1383.57 ± 761.95	24	−1.82	0.08	
Non-matriarch status	−1564.44 ± 753.95	24	−2.08	0.04	
Age × social status (grand matriarch)					
Age × matriarch status	−33.27 ± 18.42	178	1.81	0.07	
Age × non-matriarch status	−39.79 ± 18.18	178	2.19	0.03	

**Figure 4:**
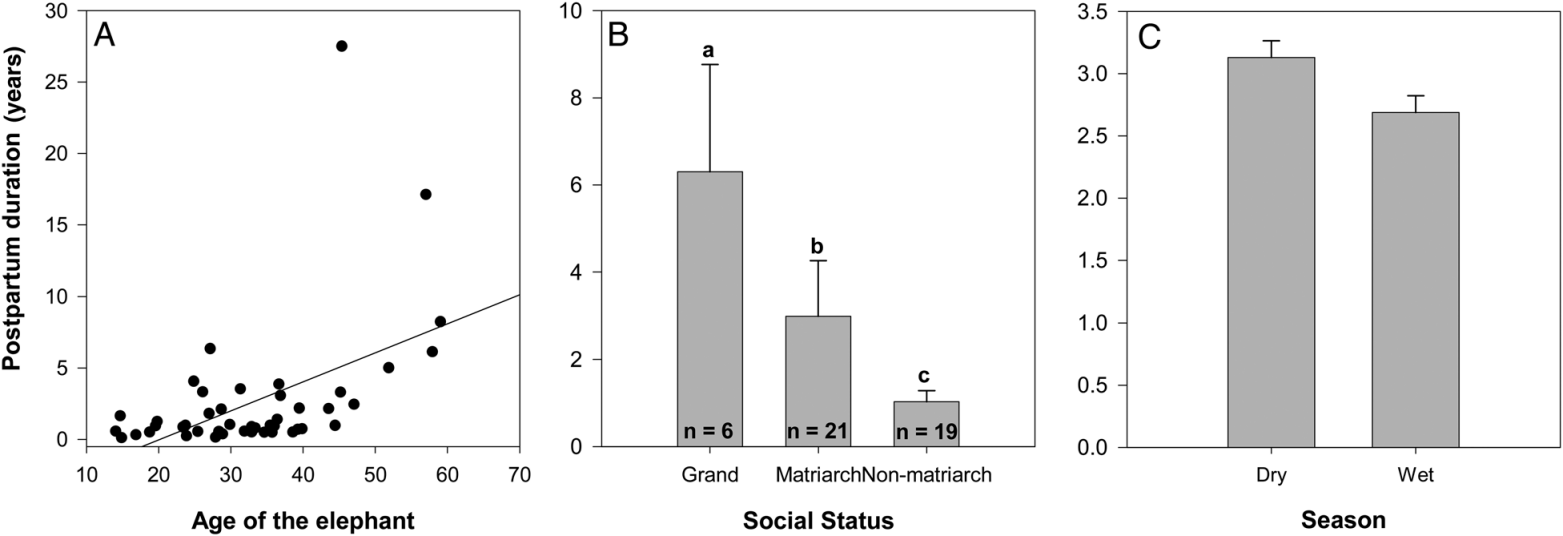
Post-partum duration in non-pregnant elephants. Relationship between post-partum duration and age (**A**), social rank (**B**), or wet/dry season (**C**) for African elephants in Addo Elephant National Park, South Africa. Superscripts designate significant differences (*P* < 0.05, Tukey's test).

Similar to the models predicting FPM concentrations, the interaction of age and social rank was included in the exploration of factors that contributed to PPD in non-pregnant elephants (Table 5). Based upon Akaike's information criteria and Bayesian information criteria, we selected the reduced model where age, social status, and season contributed to PPD. The PPD increased with the age of the female (*F*_1,359_ = 1056.11, *P* < 0.001; Fig. 4A) and was longer during the dry season in comparison to the wet season (*F*_1,359_ = 5.78, *P* = 0.02; Fig. 4C). Additionally, PPD was significantly longer (*F*_2,42_ = 24.67, *P* < 0.001; Fig. 4B) in non-pregnant, grand matriarchs than matriarchs (Tukey's test, *P* < 0.001) and non-matriarchs (Tukey's test, *P* < 0.001); PPD was also longer in matriarchs than in non-matriarchs (Tukey's test, *P* < 0.001). None of the other variables, FPM concentration of each sample, sex of the previous calf, and the total monthly precipitation at the time of sample collection, was related to PPD (Table 5).

**Table 5:** Linear mixed-effects models of factors that influence the number of post-partum years exhibited by non-pregnant female African elephants in Addo Elephant National Park

Full model	Coefficient ± SEM	d.f.	*t*-value	*P*-value	Akaike's information criteria Bayesian information criteria
Intercept	−42.88 ± 3.40	357	−12.62	<0.001	561.95
Age	0.96 ± 0.05	357	20.09	<0.001	609.79
FPM (ng/g faeces)	<0.01 ± <0.01	357	0.04	0.97	
Log_10_ precipitation (mm)	−0.04 ± 0.04	357	−0.95	0.34	
Season (dry)					
Wet season	−0.05 ± 0.04	357	−1.35	0.17	
Sex of last calf (female)					
Male calf	−0.89 ± 1.61	41	−0.57	0.58	
Social status (grand matriarch)					
Matriarch status	17.94 ± 3.71	41	4.83	<0.001	
Non-matriarch status	28.92 ± 3.73	41	7.75	<0.001	
Age × social status (grand matriarch)					
Age × matriarch status	−0.18 ± 0.06	357	−3.01	<0.01	
Age × non-matriarch status	−0.31 ± 0.07	357	−4.46	<0.001	
Reduced model	Coefficient ± SEM	d.f.	*t*-value	*P*-value	Akaike's information criteria Bayesian information criteria
Intercept	−43.60 ± 3.27	359	−13.32	<0.001	
Age	0.96 ± 0.05	359	20.36	<0.001	
Season (dry)					
Wet season	−0.06 ± 0.03	359	−1.86	0.06	
Social status (grand matriarch)	541.30				
Matriarch status	18.13 ± 3.69	42	4.91	<0.001	577.25
Non-matriarch status	29.17 ± 3.71	42	7.87	<0.001	
Age × social status (grand matriarch)					
Age × matriarch status	−0.18 ± 0.06	359	−3.06	<0.01	
Age × non-matriarch status	−0.32 ± 0.07	359	−4.51	<0.001	

## Discussion

Linear mixed-effects models indicated that social status was the most significant predictor of FPM concentrations in non-pregnant elephants within AENP. Likewise, both age and social status influenced FPM concentrations in pregnant AENP elephants. The negative relationships within each model demonstrated that grand matriarchs have lower FPM concentrations than matriarchs and non-matriarchs and that older pregnant females have lower FPM concentrations than younger pregnant elephants. For PPD, positive relationships were also demonstrated between age and social status, with non-pregnant grand matriarchs and older females having the longest intervals since the birth of their last calf. In addition, season was related to PPD; a shorter PPD was found during the wet than the dry season, because elephants were more likely to give birth in the wet season (59% of births in the present study). As our results demonstrate, because age and social status were strongly related, it is difficult to tease apart the impact of these two factors on reproductive function. Nevertheless, this is the first study to provide evidence that ovarian activity declines in older African elephants as they attain grand matriarch status.

Reproduction in female mammals is dynamic and can be affected by the interplay of multiple biotic and abiotic factors (Atsalis *et al.*, 2008). Females that survive in the wild to an advanced age are typically reproductively robust (Walker *et al.*, 2008; Finch *et al.*, 2010) and may exhibit behavioural traits that enhance their health and survivability (Walker and Herndon, 2008). In wild populations, mortality rates can be high, whether caused by predation, disease, or other factors. As a result, most females fail to reach an advanced age, which precludes the observation of high rates of reproductive senescence in wild populations (Atsalis and Margulis, 2008). Thus, a continuation of reproductive function, even at a diminished level, and survival beyond the natural fertile period is a rare event that deserves notice (Erwin and Hof, 2008). Ovarian or endocrine data on reproductive ageing have been studied in only a few mammalian species (Finch and Holmes, 2010), but all of the studies demonstrate the importance of ovarian cycling and steroidogenesis in the maintenance of female reproduction (Ottinger, 2010).

Like humans, most female mammals display a decline in fertility with advanced age (Bellino and Wise, 2003; McAuliffe and Whitehead, 2005; Kachel *et al.*, 2011). Although female African elephants in the wild have been known to reproduce into their fifties (Laws *et al.*, 1970; Smuts, 1975; Freeman *et al.*, 2009; Robinson *et al.*, 2012), a lack of detectable corpora lutea in most females over 50 years of age in Kruger National Park, South Africa indicated that they had inactive ovaries and were no longer reproductively viable (Freeman *et al.*, 2009). By 50 years of age, those females were most likely to be the matriarchs of their family or grand matriarchs of their kinship group.

As predicted, an inverse relationship between age, social status, and ovarian function (FPM) was found among the adult African elephants in AENP. The relatively low FPM concentrations and long PPD observed among the older AENP elephants, and longitudinal FPM profiles that remained at baseline in an earlier study (Freeman *et al.*, 2011), indicate that the four grand matriarchs over the age of 50 years and one matriarch (aged 44 years at study onset) may have entered reproductive senescence. Additionally, only three of the 12 elephants in this study that were ≥ 40 years of age were pregnant; two were younger grand matriarchs (39 and 43 years) and one a matriarch (38 years). Due to possible lost pregnancies (e.g. miscarriages) and females that gave birth after the study ended, the percentage of older pregnant females (25%) in AENP was probably under-estimated. However, it is likely that many of the AENP females over the age of 45 years were not pregnant and did not have functional corpora lutea, similar to the Kruger population. There is likely to be considerable individual variability in ovarian responses to ageing. Variable numbers of oocytes endowed to AENP females at birth (Finch and Holmes, 2010) could explain why some older matriarchs and grand matriarchs were still giving birth, while others had lower FPM concentrations and a longer PPD. It was somewhat unexpected that age was related to FPM concentrations only in pregnant elephants, but the results nevertheless corroborate those found among free-ranging females in Kenya (Wittemyer *et al.*, 2007b). That study did not relate FPM concentrations to social status; additional studies are needed to determine whether similar relationships exist for other elephant populations. The decrease in FPM taken together with the longer PPD with age and elevated social status among elephants ­provide physiological evidence of age-related reproductive senescence (Wittemyer *et al.*, 2007b) or menopause in elephants, as has long been suspected (Laws, 1970). A logical next step would be to examine how FPM concentrations and longer PPD in older, higher ranking elephants might be related to calf birth weights and survival, and/or milk yield.

Behavioural mechanisms may promote older female elephants reaching reproductive senescence (Laws, 1969, 1970). Senescence is an adaptive trade-off between continued reproduction and assisting kin (McAuliffe and Whitehead, 2005). In large, long-lived, highly social animals, such as elephants, cessation of reproduction before death may be selected for when the energy devoted to the care and survival of offspring can increase inclusive fitness (Hamilton, 1964). The demonstrated benefits that the social knowledge of older matriarchs impart on the family unit (McComb *et al.*, 2011) and high rates of co-operative behaviours among related females (Douglas-Hamilton, 1972; Wittemyer *et al.*, 2005; McComb *et al.*, 2011) suggest that early reproductive senescence may be selected for in elephants. Additionally, older elephants are more likely to have close relatives in their social group than young females, which increases the benefits of ceasing ­reproduction to assist close kin (Johnstone and Cant, 2010). In turn, the higher reproductive rates of kin impart fitness benefits that promote cessation of reproduction in the matriarch or grand matriarch. These social dynamics may be why reproductive success (Laws *et al.*, 1970; Smuts, 1975; Moss, 2001; Freeman *et al.*, 2009) and FPM concentrations of wild African elephants decline when females reach advanced age (>45–50 years) and matriarchal social status (e.g. become grand matriarchs).

Matriarchs and grand matriarchs also benefit the family unit by sharing information about the location of historical food sources (Gobush *et al.*, 2008). Gobush *et al.* (2008) studied the impact of the loss of old matriarchs (similar in age to grand matriarchs in the present study) on the remaining family members within a heavily poached elephant ­population of Mikumi National Park, Tanzania. Adult females without an older matriarch had lower reproductive output and higher stress levels. These studies (Gobush *et al.*, 2008; McComb *et al.*, 2011) reinforce the importance of elephant matriarchs and the benefits they can impart to their families. If cultural transmission of knowledge plays a role in the evolution of a post-reproductive lifespan, it may be through more subtle means than increasing the survival of offspring and grand offspring (Ward *et al.*, 2009). More research is required to determine how reproductive senescence is attributed to homologous physiological patterns in ovarian decline (Finch and Holmes, 2010), or the evolutionary need for kin selection or the transfer of inter-generational knowledge.

In our study, there was an environmental effect on reproductive activity. Specifically, the post-partum duration in AENP elephants was shorter during the wet season, when vegetative quality is generally higher. Enhanced vegetative quality during periods of higher rainfall can positively influence birth rates of elephants (Gough and Kerley, 2006; Foley and Faust, 2010). Addo Elephant National Par elephants have higher birth rates during wet than dry years (Gough and Kerley, 2006). Although elephants can give birth year round, most (33 of 80 births) during the course of our study occurred during AENP rainfall peaks in October–November and February–March. Giving birth in the wet season ensures that females are in optimal body condition as lactational demands increase (Laws and Parker, 1968). Given that elephants typically nurse a calf until the next one is born, most juveniles are weaned during the wet season when vegetative quality can compensate for the calories no longer gained from milk. Poor nutritional quality during the dry season causes a decline in body condition, which may negatively impact the success of implantations and early pregnancies (Laws and Parker, 1968; Foley *et al.*, 2001) as well as contributing to lower FPM concentrations for both pregnant (Foley *et al.*, 2001; Wittemyer *et al.*, 2007b) and non-pregnant elephants (Wittemyer *et al.*, 2007b). Females that do not cycle because of a lack of available browse and poor body condition during the dry season are unlikely to conceive. Such natural regulation of oestrous cycle activity may help to regulate the timing of conceptions and births. Reproductive seasonality has been documented for populations of elephants in Kenya, South Africa, Tanzania, Uganda, and Zambia (Laws and Parker, 1968; Laws, 1969; Smuts, 1975; Poole, 1989; Stuart-Hill *et al.*, 1993; Foley *et al.*, 2001; Wittemyer *et al.*, 2007a; Freeman *et al.*, 2009; Foley and Faust, 2010).

We expected to find seasonal variability in FPM concentrations, similar to the studies in Kenya (Wittemyer *et al.*, 2007b) and Tanzania (Foley *et al.*, 2001); however, no such relationship was found between precipitation or wet/dry ­season and FPM concentrations in pregnant or non-pregnant elephants in AENP. Similar to these two populations (Wittemyer *et al.*, 2007a; Foley and Faust, 2010), elephant birth rates in AENP are positively correlated with rainfall in the year of conception (Gough and Kerley, 2006). Unlike those populations, AENP elephants have access to drought-resistant vegetation (Stuart-Hill and Aucamp, 1993), artificial water sources, and rainfall year round (Gough and Kerley, 2006), and show very little variation in body condition throughout the year (J. M. Meyer, personal observation). The consistent condition of the elephants year round may explain why no differences in FPM concentrations with respect to precipitation or season were found in the AENP population.

The access to water and the consistent body condition of the elephants in AENP may also contribute to their relatively high population growth rate (5.8 ± 3.1%; Gough and Kerley, 2006). The average IPI found in the present study (1.88 ± 0.55 months) would produce a similar inter-calving interval (3.3 ± 0.8 years) to that reported previously for AENP (Gough and Kerley, 2006), assuming a 22 month gestation (Laws, 1969; Wittemyer *et al.*, 2007b). One third of the pregnant females within our sample AENP population (*n* = 9) conceived within a year of giving birth; four of those females lost their calf and appear to have re-cycled and conceived shortly thereafter. In spite of the rapidly expanding population and a density that has exceeded recommendations for 50 years (Kerley *et al.*, 2006), the elephant population in AENP has yet to experience any density-dependent regulation (Gough and Kerley, 2006).

### Conclusions

Our study advances knowledge about reproductive physiology in free-ranging elephants by providing evidence of a relationship between older females obtaining the highest social status within their family and declines in FPM concentrations and increases in PPD. Reproductive senescence contributes to a post-reproductive lifespan for elephant matriarchs and grand matriarchs, when they may provide survival benefits to their offspring and extended family members because of the knowledge they impart. However, a post-reproductive lifespan will only evolve if the indirect fitness benefits that accrue outweigh additional attempts at direct fitness output once females reach advanced age (>45 years) and high social rank. Owing to the high density of elephants within AENP (Kerley and Landman, 2006), there may be further selective pressures on the oldest females to stop adding more individuals to the population. The present study provides further evidence of a decline in reproductive success with advanced age in elephants. More research on other populations with larger numbers of matriarchs and grand matriarchs, and lower population densities, is needed to determine whether selective pressures have led to the evolution of menopause in female elephants, as suggested by Laws (1969).

Knowledge about reproductive physiology of high-ranking females can provide managers with biological data to identify the best candidates for policy decisions when population growth needs to be regulated. Elephant over-population is a growing problem in some areas of Africa (Owen-Smith *et al.*, 2006), and the density of elephants in AENP has exceeded recommended limits, by up to 8-fold, for 50 years (Kerley and Landman, 2006). Although the AENP elephants have a high population growth rate, which is coupled with low juvenile and adult mortality, these demographic factors are not density dependent in the AENP population (Gough and Kerley, 2006). The influence of the AENP elephants on the succulent thicket vegetation is well documented (Lombard *et al.*, 2001; Kerley and Landman, 2006; Landman *et al.*, 2008). It is predicted that the elephant population in AENP will continue to grow and reproduce at a high rate until the vegetative resources are irreversibly depleted (Gough and Kerley, 2006). Although fluctuating elephant populations can be beneficial to biodiversity (Whyte *et al.*, 1999), it is not known whether uncontrolled growth will irreversibly harm it (Dickson and Adams, 2009) or be detrimental to ecosystem functioning in the long term (Landman *et al.*, 2012). Furthermore, competition with elephants for dwindling resources in AENP appears to be impacting the health (Aronoff JT, Santymire RM, Freeman EW, Meyer J, Gillespie TR, unpublished), foraging opportunities and diet (Landman and Kerley, 2013; Landman *et al.*, 2013), and activity patterns (Tambling CJ, Meyer J, Minnie L, Freeman EW, Santymire RM, Addendorf J, Kerley GIH, unpublished) of the critically endangered black rhinoceros (*Diceros bicornis bicornis*). Thus, for the sake of other animal and plant species, growth of the AENP elephant population may need to be controlled before it naturally reaches carrying capacity (Whyte, 2001; Gough and Kerley, 2006).

There is much debate about the best tools to manage growing elephant populations (Owen-Smith *et al.*, 2006) and whether scientific data prove that they need to be regulated at all (Dickson and Adams, 2009). Historically, elephant populations in South Africa were controlled through culling, and in 2008 South Africa voted to resume this practice (Dickson and Adams, 2009). Contraception is another possible means to control elephant populations, and its use has been tested repeatedly in South Africa (Stetter *et al.*, 2006; Kerley *et al.*, 2007; Fayrer-Hosken *et al.*, 1999; Druce *et al.*, 2011, 2013). Translocations, reintroductions, and the creation of mega-transfrontier parks (van Aarde *et al.*, 2006, 2007) are also proposed as management tools. Regardless of the methods selected, physiological data (Cooke *et al.*, 2013), such as those gained through non-invasive endocrine monitoring, can provide critical information to population managers. For instance, monitoring of hormone patterns can demonstrate the efficacy of culling and contraceptives (Wasser *et al.*, 1996; Foley *et al.*, 2001) on reproductive function, and identify the best candidates for translocation (Freeman *et al.*, 2011; Cooke *et al.*, 2013). Additionally, measures of both reproductive (e.g. FPM) and stress hormones (e.g. glucocorticoids) can be used to assess the impact of human disturbance (e.g. poaching and habitat fragmentation) and environmental change on overall reproductive health and animal welfare, with implications for conservation management.

With our growing understanding of the relationships among age, social status, and progestagen concentrations, the endocrine status of females should be monitored prior to selecting them for any population-control programmes. Due to the high costs and controversial nature of most policies for regulating elephant populations (Dickson and Adams, 2009), knowledge of the reproductive status of individuals in the population would enhance the efficacy of these management decisions. For instance, discovering that a female is no longer reproductively viable would eliminate her as a candidate for contraception. In particular, grand matriarchs should be excluded from consideration for culling, contraception, and/or translocation without assessing their reproductive status first, because of the important role they play in elephant society and the likelihood that these females are no longer cycling.
